# Interobserver Agreement in Immunohistochemical Evaluation of Folate Receptor Alpha (FRα) in Ovarian Cancer: A Multicentre Study

**DOI:** 10.3390/ijms26167687

**Published:** 2025-08-08

**Authors:** Gian Franco Zannoni, Giuseppe Angelico, Antonio d’Amati, Nicoletta D’Alessandris, Giulia Scaglione, Belen Padial Urtueta, Gerardo Ferrara, Anna Caliò, Paola Campisi, Antonio De Leo, Elena Guerini Rocco, Martina Iuzzolino, Lucia Lerda, Biagio Paolini, Alessandra Punzi, Mattia Vinci, Giancarlo Troncone, Angela Santoro

**Affiliations:** 1 Pathology Unit, Department of Woman and Child’s Health and Public Health Sciences, Fondazione Policlinico Universitario Agostino Gemelli IRCCS, Largo A. Gemelli 8, 00168 Rome, Italybelen.padialurtueta@policlinicogemelli.it (B.P.U.); angela.santoro@policlinicogemelli.it (A.S.); 2 Pathology Institute, Catholic University of Sacred Heart, 00168 Rome, Italy; 3 Department of Medicine and Surgery, Kore University of Enna, 94100 Enna, Italy; 4 Anatomic Pathology and Cytopathology Unit, G. Pascale Foundation National Cancer Institute IRCCS, 80131 Naples, Italy; gerardo.ferrara@istitutotumori.na.it; 5 Department of Diagnostics and Public Health, Section of Pathology, University of Verona, 37134 Verona, Italy; 6 Department of Pathology, Umberto I Hospital, Largo Turati 62, 10128 Turin, Italy; 7 Department of Medical and Surgical Sciences (DIMEC), University of Bologna, 40138 Bologna, Italy; 8 Department of Oncology and Hemato-Oncology, University of Milano, 20122 Milan, Italy; 9 Division of Pathology, IEO European Institute of Oncology IRCCS, 20141 Milan, Italy; 10 Department of Pathology, IRCCS Humanitas Research Hospital, Via Manzoni 56, 20089 Rozzano, MI, Italy; 11 Department of Biomedical Sciences, Humanitas University, Via Rita Levi Montalcini 4, 20072 Pieve Emanuele, MI, Italy; 12 Pathology Unit, Centro di Riferimento Oncologico di Aviano (C.R.O.) IRCCS, 33081 Aviano, Italy; 13 Department of Gynecologic Oncology, Fondazione IRCCS Istituto Nazionale Dei Tumori di Milano, 20133 Milan, Italy; biagio.paolini@istitutotumori.mi.it; 14 Pathology Unit, IRCCS National Cancer Institute “Giovanni Paolo II”, 65 Orazio Flacco St., 70124 Bari, Italy; 15 Department of Pathology, Treviso Regional Hospital, 31100 Treviso, Italy; 16 Department of Public Health, University of Naples “Federico II”, 80138 Naples, Italy

**Keywords:** ovarian cancer, folate receptor alpha, high-grade serous ovarian carcinoma, VENTANA FOLR1, immunohistochemistry

## Abstract

Folate receptor alpha (FRα) is a high-affinity folate transporter overexpressed in various epithelial malignancies, particularly high-grade serous ovarian carcinoma. Given its restricted expression in normal tissues and accessibility in tumors, FRα is an emerging therapeutic target. Immunohistochemistry (IHC) is the standard method for FRα assessment; however, interpretation is semi-quantitative and prone to interobserver variability. This study aimed to evaluate interobserver agreement among 12 pathologists in the IHC assessment of FRα in ovarian cancer, focusing on internal control adequacy, staining intensity, and the percentage of FRα-positive tumor cells. Thirty-seven high-grade serous ovarian carcinoma cases were stained using the VENTANA FOLR1 (FOLR1-2.1) RxDx Assay. A reference panel of four expert pathologists established consensus diagnoses. Twelve pathologists independently assessed the slides, recording internal control adequacy, staining intensity (positive vs. negative), and percentage of FRα-positive tumor cells. Interobserver agreement was measured using Fleiss’ kappa and intraclass correlation coefficient (ICC). Agreement on internal control adequacy was almost perfect (κ = 0.84). Substantial agreement was observed for staining intensity (κ = 0.76), while percentage estimation showed almost perfect concordance (ICC = 0.89). Discrepancies were primarily confined to borderline cases (65–85% positivity) and tumors with intermediate staining, reflecting interpretive challenges near clinical decision thresholds. Pathologists demonstrated high reproducibility in FRα IHC assessment, particularly in estimating percentage positivity and control adequacy. These findings support the clinical utility of FRα IHC but underscore the need for standardized scoring criteria and potential integration of digital tools to enhance consistency, especially in borderline cases.

## 1. Introduction

Folate receptor alpha (FRα) is a glycosylphosphatidylinositol-anchored cell surface protein that functions as a high-affinity, low-throughput folate transporter, facilitating folate uptake via receptor-mediated endocytosis to support one-carbon metabolism, DNA synthesis, and cellular proliferation [1]. FRα is frequently overexpressed in a range of epithelial malignancies, including approximately 35–70% of breast cancers, 15–75% of lung cancers, 20–50% of endometrial cancers, and 75–90% of ovarian cancers [2,3,4]. Among ovarian cancers, high-grade serous carcinoma demonstrates the highest frequency of FRα overexpression, reported in roughly 60–85% of cases [2,3,4,5,6]. These estimates vary due to differences in detection methods, antibody clones, sample sizes, and scoring criteria across studies [2,3,4,5,6].

Recent advances highlight the importance of integrating molecular profiling into ovarian cancer management. BRCA1/2 mutations in high-grade serous ovarian carcinoma (HGSOC) have established the value of biomarker-driven therapies [7]. Similarly, FRα expression offers a promising target for patient stratification. Beyond biology, the psychological burden of advanced or recurrent disease can impact quality of life and care adherence. Improving diagnostic accuracy and enabling personalized therapy may also support better patient-reported outcomes, including emotional well-being and perceived disease control [7].

Due to its selective expression in malignant tissues and limited distribution in normal adult tissues, FRα has emerged as a promising therapeutic target, with several FRα-directed agents currently under clinical investigation [8,9,10,11,12,13,14].

Immunohistochemistry (IHC) is the standard method for evaluating FRα expression in clinical and research settings, guiding eligibility for FRα-targeted therapies [8,9,10,11,12,13,14]. However, IHC scoring of FRα remains inherently semi-quantitative and susceptible to interobserver variability [15,16,17,18]. Most commonly adopted IHC scoring systems rely on the assessment of both the intensity and the extent of tumor cell membrane staining [15,16,17,18]. The scoring criteria used in clinical trials, and increasingly referenced in routine practice, classify tumors as “FRα-high” when ≥75% of viable tumor cells demonstrate at least moderate (2+) membranous staining [15,16,17,18]. While this threshold has shown clinical utility, particularly in patient selection for FRα-targeted treatments, its reproducibility across different observers and institutions remains insufficiently characterized [15,16,17,18].

In routine diagnostic practice, interpretation of FRα IHC can be influenced by a range of technical and subjective factors. These include variability in staining protocols, inconsistencies in the quality or presence of internal control tissue (typically represented by benign tubal epithelium), and differences in individual pathologist experience or interpretive thresholds [15,16,17,18]. Moreover, the distinction between membranous and cytoplasmic staining, and the identification of partial or heterogeneous staining patterns, may further complicate assessment, particularly in borderline or equivocal cases [15,16,17,18].

Given the clinical implications of FRα status for patient stratification and therapeutic decision-making, it is essential to establish the reliability and reproducibility of FRα IHC evaluation in real-world pathology settings. A robust understanding of interobserver agreement can help inform the standardization of scoring guidelines and support the integration of FRα testing into routine diagnostic workflows.

This study aims to evaluate interobserver variability in the assessment of FRα IHC in ovarian cancer among a group of 12 pathologists. Using a cohort of 37 ovarian cancer cases with accompanying internal control tissue, selected from institutional archives based on histological diagnosis, adequate tumor representation, and technically acceptable FRα immunostaining, we focus on three key parameters: (1) adequacy of internal control tissue, (2) FRα positivity versus negativity, and (3) estimation of the percentage of tumor cells exhibiting FRα staining. Through this analysis, we seek to quantify agreement across observers and identify areas where interpretive consistency may be improved.

This study builds upon the conceptual framework outlined in our previous review on FRα in ovarian cancer [19], but differs substantially in scope and methodology. The present investigation is based on a distinct cohort of ovarian carcinoma cases and provides prospective, observer-based validation of FRα immunoscoring, which was not addressed in the earlier work.

## 2. Results

### 2.1. Internal Control Adequacy

The reference panel classified 36 out of 37 cases (97.3%) as having adequate internal controls, based on the presence of fallopian tube epithelium predominantly displaying moderate (2+) circumferential membranous staining, with negative stromal staining. One case was defined as inadequate due to the absence of recognizable tubal epithelium or the presence of non-specific/background staining.

Observers demonstrated consistently high agreement in assessing internal control adequacy. In 35 of 37 cases (94.6%), all 12 observers unanimously classified the internal control as adequate. One case (2.7%) was unanimously judged inadequate due to the absence of recognizable tubal epithelium or the presence of non-specific/background staining. Another case (2.7%) received discordant evaluations, with one or more observers considering the control inadequate due to weak, incomplete, or absent staining; however, the reference panel had classified the control tissue in this case as adequate. The Fleiss’ kappa statistic for interobserver agreement on internal control adequacy was 0.84, indicating almost perfect agreement according to the Landis and Koch scale. 

### 2.2. FRα Staining Intensity in Tumor Cells

Based on the reference panel assessment, 27 out of 37 cases (73%) demonstrated moderate (2+) and/or strong (3+) membranous FRα staining and were classified as positive for staining intensity. The remaining 10 cases (27%) were categorized as negative, showing absent (0) or weak (1+) staining.

Full agreement among all 12 observers regarding staining intensity (positive vs. negative) was achieved in 31 cases (83.8%). The remaining six cases (16.2%) showed discordance among observers. Among these discordant cases, four were classified as positive by the reference panel but showed variability among observers, typically due to heterogeneous staining patterns or staining near the positivity threshold. In the other two cases, which were negative according to the reference panel, weak focal staining led some observers to incorrectly classify them as positive. The Fleiss’ kappa value for staining intensity assessment was 0.76, indicating substantial interobserver agreement.

### 2.3. Percentage of FRα-Positive Tumor Cells

According to the reference panel evaluation, the distribution of FRα expression across the 37 ovarian cancer cases encompassed the full spectrum of staining extent. Five cases (13.5%) exhibited absent or minimal staining, falling within the 0–5% range, and were classified as negative. Three cases (8.1%) showed weak and focal staining involving a limited subset of tumor cells, corresponding to the 6–15% range. Four cases (10.8%) demonstrated low-level but definite membranous staining and were placed in the 16–30% category, while another three cases (8.1%) exhibited more heterogeneous patterns and were assigned to the 31–45% range. Intermediate FRα expression, involving 46–64% of tumor cells, was observed in four cases (10.8%), while more extensive staining, ranging from 65% to 85% (borderline cases), was identified in nine cases (24.3%). Finally, nine additional cases (24.3%) displayed near-complete or complete membranous positivity, with 86–100% of tumor cells stained for FRα.

Quantitative estimation of the percentage of FRα-positive tumor cells showed almost perfect agreement among the 12 observers. The calculated intraclass correlation coefficient (ICC) for this continuous variable was 0.89, with a 95% confidence interval of 0.83 to 0.94, indicating a high level of agreement. The highest concordance was observed in tumors with either extensive and uniform staining (86–100% range) or minimal expression (0–5% range), where observer estimates typically clustered within ±5–10% of the group median. Similarly, cases falling within the 65–85% interval (borderline cases) also showed strong agreement, although slightly greater variability was noted, likely due to more heterogeneous staining in some tumors. The greatest discrepancies were particularly evident among tumors exhibiting intermediate levels of FRα expression, with differences of up to 20 percentage points between observers. In fact, tumors within the 31–45% and 46–64% ranges exhibited greater interobserver variation, reflecting the interpretive challenges posed by patchy or focal staining patterns. Despite this variability, the overall agreement remained high, underscoring the reproducibility of FRα percentage estimation, particularly at the extremes of expression. 

In summary, the interobserver agreement for internal control adequacy was almost perfect (Fleiss’ kappa = 0.84). Agreement on FRα staining intensity (positive vs. negative) was substantial (Fleiss’ kappa = 0.76), while the quantification of the percentage of FRα-positive tumor cells demonstrated almost perfect agreement (ICC = 0.89; 95% CI: 0.83–0.94). These results indicate strong consistency among pathologists in evaluating all key parameters.

### 2.4. Overall FRα Positivity and Analysis of Borderline and Discordant Cases

**Figure 1:**
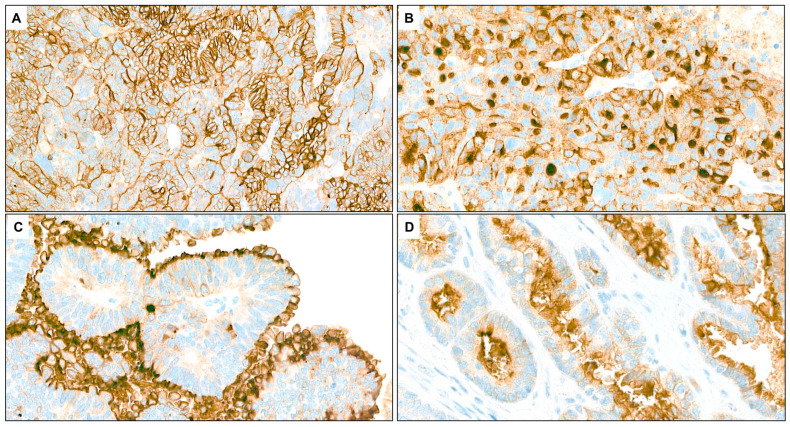
Immunohistochemical determination of folate receptor 1 status. (**A**) Ovarian High-Grade Serous Carcinoma showing strong (3+) FOLR1 membrane positivity in >75% of tumor cells (H&E, 20×). (**B**) Ovarian High-Grade Serous Carcinoma showing circumferential and dot-like membranous staining of strong intensity (3+) in >75% of tumor cells (H&E, 20×). (**C**,**D**) Two examples of ovarian High-Grade Serous Carcinoma showing apical: the FOLR1 protein is localized to the apical (top) surface of neoplastic cells (H&E, 20×).

Based on the reference panel evaluation, tumors were considered positive for FRα expression when ≥75% of tumor cells demonstrated moderate (2+) or strong (3+) membranous staining, including circumferential, apical, and dot-like patterns (Figure 1) [19,20]. Borderline cases were defined as tumors exhibiting 65–85% of neoplastic cells with 2+/3+ membranous staining, encompassing both circumferential, apical, and dot-like patterns [20,21]. Applying these criteria, the reference panel identified 9 borderline cases among the 37 analyzed tumors. These cases showed strong agreement among the observers, although slightly greater variability was noted, likely due to more heterogeneous staining in some tumors.

**Figure 2:**
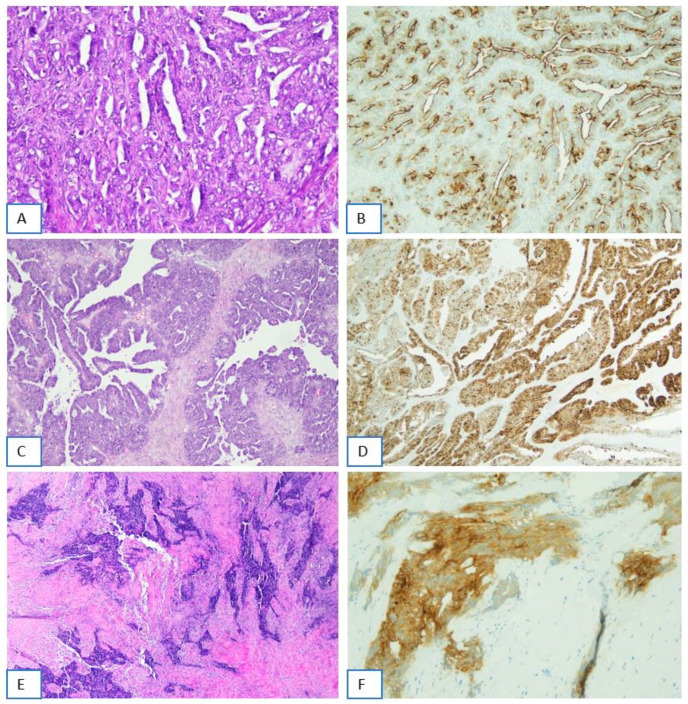
Immunohistochemical determination of folate receptor 1 status: examples of discordant cases. (**A,B**) Ovarian high-grade serous carcinoma showing FOLR1 membrane positivity (score 2+/3+) in 70% of tumor cells. This case has been considered borderline (65–85% of cells with a score of 2+ or 3+ positivity) ((**A**) H&E, 20×; (**B**) FOLR1-2.1 antibody on Benckmark Ultra platform, LSAB-HRP, 10×). (**C**,**D**) Another example falling within the borderline category: ovarian high-grade serous carcinoma showing FOLR1 membrane positivity (score 2+/3+) in 70% of tumor cells ((**C**) H&E, 10×; (**D**) Ventana FOLR1 RxDx Assay—FOLR1-2.1 antibody on (**E**,**F**) peritoneal metastasis of ovarian high-grade serous carcinoma showing FOLR1 membrane positivity not adequately evaluable, due to the diffuse crushing or heat-generated (cautery) artifacts ((**E**) H&E, 4×; (**F**) Ventana FOLR1 RxDx Assay—FOLR1-2.1 antibody on Benckmark Ultra platform, LSAB-HRP, 20×).

**Figure 3:**
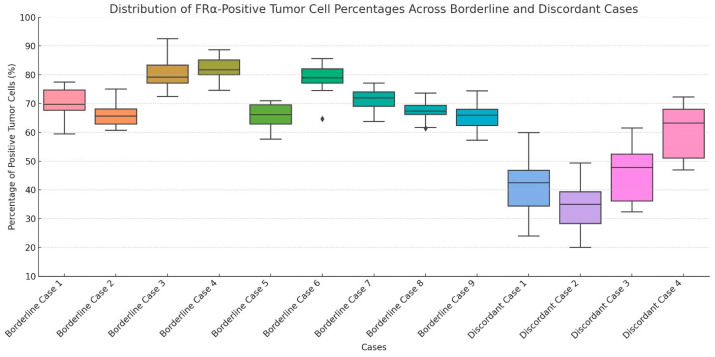
Immunohistochemical determination of folate receptor 1 status: examples of discordant cases. Distribution of estimated FRα-positive tumor cell percentages across borderline and discordant cases. Borderline cases were defined as tumors with 65–85% of neoplastic cells exhibiting 2+/3+ membranous staining, while discordant cases included tumors within the 31–45% and 46–64% percentage ranges that showed significant interobserver variability. Each boxplot represents the range, median, and variability of observer estimates for a given case.

In addition to borderline cases, discordant cases were also recognized among tumors exhibiting FRα-positive tumor cell percentages within the 31–45% and 46–64% intervals. These tumors demonstrated significant interobserver variability in the estimation of FRα-positive tumor cell percentages, with differences reaching up to 20 percentage points in some instances. In total, 4 discordant cases were identified based on their marked interpretive variability across observers. Representative examples of discordant cases are shown in Figure 2. Distribution of estimated FRα-positive tumor cell percentages across borderline and discordant cases is shown in Figure 3.

**Figure 4:**
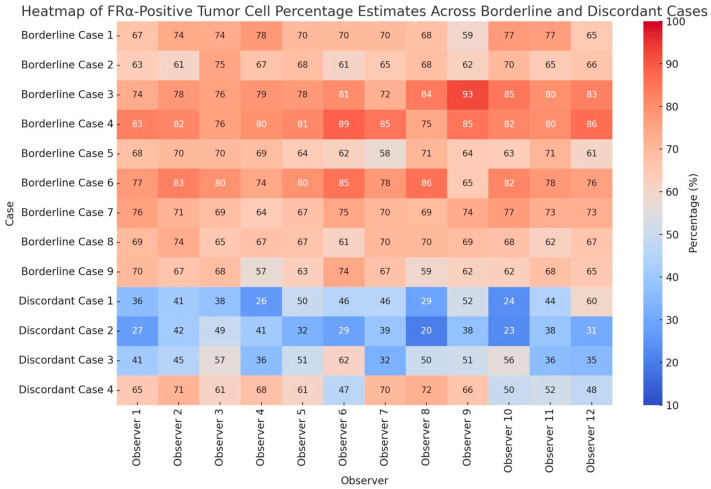
Heatmap of individual observer estimates of FRα-positive tumor cell percentages across borderline and discordant cases. Borderline cases were defined based on a 65–85% staining threshold with 2+/3+ intensity, and discordant cases were selected among tumors showing marked variability in the 31–45% and 46–64% percentage intervals. Values highlight interobserver variability patterns.

Individual observer estimates of FRα-positive tumor cell percentages across borderline and discordant cases are shown in Figure 4.

Despite these interpretive challenges, the majority of observers applied internal evaluation criteria consistently across the cohort. High-expression tumors, particularly those with homogeneous strong staining in more than 90% of tumor cells, and low-expression tumors with weak or absent staining involving less than 25% of tumor cells, showed minimal variability among observers.

## 3. Discussion

This study assessed the interobserver agreement in the immunohistochemical (IHC) evaluation of folate receptor alpha (FRα) expression in ovarian cancer across a cohort of 12 pathologists reviewing 37 cases. To our knowledge, this is among the first studies to specifically quantify diagnostic concordance in FRα IHC interpretation using real-world pathology practice conditions. Our findings indicate that pathologists can evaluate FRα expression with a high degree of reliability, particularly when it comes to assessing internal control adequacy and estimating the percentage of FRα-positive tumor cells. However, some interpretive variability was observed in binary classification decisions, especially in borderline or equivocal cases, underscoring the need for standardization in clinical practice [17,18,19,20].

The biological and clinical importance of FRα in ovarian cancer has been well established [1,2,3,4,5,6]. FRα is overexpressed in the majority of high-grade serous ovarian carcinomas and serves not only as a diagnostic and prognostic biomarker but also as a therapeutic target for antibody–drug conjugates, such as mirvetuximab soravtansine [6,7,8,9,10,11,12,13]. The efficacy of these targeted therapies is closely related to the level and distribution of FRα expression, necessitating accurate and reproducible IHC-based assessment [6,7,8,9,10,11,12,13,14]. Our results support the robustness of FRα as a biomarker, as evidenced by the strong interobserver agreement, particularly in estimating the percentage of tumor cells stained (ICC = 0.89). This finding is reassuring, as many current clinical trials and drug approvals rely on percentage thresholds (commonly ≥75%) to determine eligibility for FRα-targeted therapy [8,9,10,11,12,13,14]. The evaluation of internal control tissue is a crucial first step in IHC interpretation, ensuring that staining was technically successful. Our study demonstrates excellent reproducibility in this area (Fleiss’ kappa = 0.84), likely attributable to the distinct morphology and reliable staining of fallopian tube epithelium. This supports the inclusion of tubal tissue on FRα IHC slides as an internal quality control measure in diagnostic protocols.

Agreement on the binary classification of FRα positivity or negativity was substantial (kappa = 0.76), though notably lower than agreement on control adequacy or percentage estimation. Discrepancies in binary classification were concentrated in tumors with intermediate staining (approximately 50–80% of cells positive) or with weak membranous staining. These are the same cases most likely to present interpretive challenges in clinical practice, where classification decisions can directly influence patient eligibility for FRα-targeted therapy. The reliance on semi-quantitative scoring systems, subjective intensity grading, and lack of digital standardization likely contribute to this variability. Notably, the cases that generated the most disagreement fell near the 75% cutoff point used in clinical trials such as MIRASOL [21]. These findings suggest that interpretive ambiguity around such thresholds may lead to inconsistent classification in the absence of strict criteria or centralized review.

There is currently no universally accepted scoring system for FRα IHC in routine diagnostic practice, though clinical trials have converged on a simplified binary scheme based on the percentage of viable tumor cells showing ≥2+ membranous staining [8,9,10,11,12,13,14,15,16].

Our findings reinforce the utility of this scheme but also emphasize that scoring performance may improve significantly with proper training and calibration of observers. Incorporating digital pathology tools or automated image analysis systems may further improve objectivity, especially in borderline cases. Alternatively, implementing a three-tier scoring system (e.g., FRα-high, FRα-intermediate, FRα-negative) could capture more nuance in expression patterns while mitigating the binary cutoff dilemma.

The strengths of this study include its multi-observer design, the use of a standardized FRα IHC protocol, and the inclusion of a well-characterized internal control. However, there are limitations. The study cohort was relatively small and enriched for high-grade serous carcinoma, which may limit generalizability to rarer histologic subtypes. Additionally, the variability in pathologist experience and lack of formal pre-review calibration could have contributed to some of the observed discrepancies, although this also makes the results reflective of real-world conditions.

While the lack of pre-assessment training provided insight into interpretative variability, future studies would benefit from structured calibration efforts. These may include pre-evaluation consensus training, digital reference panels, or AI-assisted annotation tools to improve interobserver agreement and facilitate reproducibility in clinical application.

Finally, staining was assessed visually and manually; future studies using digital pathology and artificial intelligence may provide a more granular assessment of reproducibility and accuracy.

In conclusion, our results demonstrate a high level of interobserver agreement in FRα IHC interpretation among pathologists, particularly for percentage estimation and control tissue adequacy. While binary classification showed substantial concordance, cases near the threshold of positivity present interpretive challenges.

In clinical practice, cases with 2+/3+ FOLR1 membranous staining in 65–85% of tumor cells fall within a borderline zone that may impact therapeutic eligibility for FRα-targeted agents. Given that most protocols adopt a ≥75% threshold, such cases may warrant additional review, including repeat testing or consensus review, to ensure appropriate patient selection.

Our findings highlight the necessity of clear scoring criteria, suggest the incorporation of digital aids, and advocate for a more nuanced classification system to support clinical decision-making for FRα-targeted therapies.

In this regard, several digital pathology platforms are emerging as promising tools to improve the reproducibility of FOLR1 scoring. Deep learning-based systems such as HALO AI, PathAI, and Visiopharm can accurately segment tumor areas and quantify membranous staining [22,23]. Additionally, rule-based tools like QuPath allow for customizable algorithmic thresholding to calculate the percentage of tumor cells with 2+/3+ FOLR1 expression [24]. Integration of these technologies may support more standardized therapeutic decision-making, particularly in borderline cases.

Therefore, in future phases of this project, we aim to incorporate AI-assisted tools for digital scoring of FRα expression using whole-slide imaging. These approaches have already demonstrated utility in assessing HER2, PD-L1, and Ki-67 in various tumor types and could similarly enhance the diagnostic value of FRα evaluation.

While prior reviews, including our own, have summarized the biological and clinical relevance of FRα, the current study is the first to apply a prospective, multi-observer scoring protocol to a newly curated set of cases [20].

As FRα continues to gain relevance in ovarian cancer management, ensuring reproducibility in its assessment will be essential for its successful clinical implementation.

## 4. Materials and Methods

### 4.1. Case Selection and Immunohistochemistry

A total of 37 formalin-fixed, paraffin-embedded (FFPE) high-grade serous ovarian carcinoma (HGSOC) cases were retrospectively selected from the institutional archives of the. Case selection was performed independently by three experienced gynecologic pathologists based on the following criteria: (i) confirmed histopathological diagnosis of HGSOC according to WHO 2020 classification, (ii) sufficient viable tumor tissue on slide, and (iii) technically adequate FRα immunostaining. Only cases with optimal internal control tissue (normal-appearing fallopian tube epithelium) on the same slide were included to validate staining quality.

Immunohistochemistry for folate receptor alpha (FRα) was performed using the VENTANA FOLR1 (FOLR1-2.1) RxDx Assay (Roche Diagnostics, Basel, Switzerland), run on the VENTANA BenchMark ULTRA platform [15,16,18]. The assay uses a monoclonal rabbit anti-human FRα antibody (clone FOLR1-2.1) pre-diluted and optimized by the manufacturer [15,16,18]. The procedure was conducted following the manufacturer’s standardized protocol, which includes antigen retrieval using Cell Conditioning 1 (CC1) for 64 minutes, followed by incubation with the primary antibody for 16 minutes at 37 °C. Detection was achieved using the OptiView DAB IHC Detection Kit [15,16,18].

Negative controls were performed by omitting the primary antibody, while positive controls consisted of normal fallopian tube epithelium, known to express FRα on the apical membrane of ciliated epithelial cells [15,16,18]. Each slide was assessed for both control and tumor staining to confirm technical adequacy and biological relevance.

FRα expression was assessed semi-quantitatively across the entire tumor section based on: (1) Staining intensity: graded as 0 (no staining), 1+ (weak), 2+ (moderate), or 3+ (strong); (2) Percentage of tumor cells stained at each intensity level; 3) Subcellular localization: only membranous staining was considered specific and scored, whereas cytoplasmic or nuclear reactivity was disregarded as non-specific background [15,16,18].

### 4.2. Reference Evaluation

A panel of four experienced gynecological pathologists independently reviewed all 37 immunostained slides to establish a reference diagnosis. Their assessment included (i) adequacy of internal control tissue (fallopian tube epithelium); (ii) FRα staining intensity in tumor cells (negative or positive); and (iii) percentage of tumor cells exhibiting membranous FRα staining (continuous variable). The consensus evaluation of the panel served as the diagnostic gold standard for subsequent interobserver comparison.

### 4.3. Observer Evaluation

Twelve pathologists, including general surgical and gynecological subspecialists with varying levels of experience, independently reviewed all 37 cases using digital or glass slide formats. Observers were blinded to both the reference panel evaluation and to each other’s assessments. A formal theoretical session (3 h of lectures) was conducted; however, no training session or consensus discussion was held prior to the evaluation phase. Each observer was asked to record the same parameters evaluated by the reference panel: (i) adequacy of internal control tissue (fallopian tube epithelium); (ii) FRα staining intensity in tumor cells (negative or positive); (iii) percentage of tumor cells exhibiting membranous FRα staining (continuous variable).

### 4.4. Assessment of Internal Control Adequacy

The internal control was assessed based on the presence and staining quality of fallopian tube epithelium on each slide. The internal control was considered adequate if fallopian tube epithelium was present on the slide and exhibited circumferential membranous staining of moderate intensity (2+), with or without intense apical staining (3+). The accompanying stromal tissue was required to show no staining (0) [18,19]. Adequacy of internal control tissue was independently assessed by all observers prior to tumor evaluation.

### 4.5. Assessment of FRα Staining Intensity in Tumor Cells

Only membranous staining, whether complete or incomplete, was considered for intensity scoring, in line with recommendations for FRα-targeted therapy eligibility [18,19]. Tumor staining intensity was categorized based on membranous immunoreactivity as negative (absent staining 0 or weak staining 1+) or positive (moderate staining 2+ or strong staining 3+) [18,19].

### 4.6. Assessment of the Percentage of FRα-Positive Tumor Cells

For each case, observers independently estimated the percentage of tumor cells exhibiting membranous FRα staining, regardless of intensity. Percentage estimates were recorded as continuous variables and grouped into predefined intervals (0–5%, 6–15%, 16–30%, 31–45%, 46–64%, 65–85%, and 86–100%).

### 4.7. Assessment of Overall FRα Positivity and Definition of Borderline Cases

Tumors were considered positive for FRα expression when ≥75% of tumor cells exhibited moderate (2+) or strong (3+) membranous staining, including apical or dot-like patterns, in accordance with established clinical thresholds for eligibility to FRα-targeted therapies [18,19].

Borderline cases were defined as tumors exhibiting moderate (2+) or strong (3+) membranous staining in 65–85% of tumor cells, corresponding to a range within ±10% of the established cutoff for FRα positivity, and thus positioned at the interface between positive and negative status [18,19].

Such borderline cases were identified because minor differences in the estimation of stained tumor cell percentage could lead to divergent clinical classifications regarding eligibility for FRα-targeted therapies [18,19].

## Data Availability

Manuscript data are available from the corresponding author upon reasonable request.
